# A novel frameshift *RPL15* gene variant associated with hydrops fetalis and perinatal death

**DOI:** 10.3389/fgene.2026.1924019

**Published:** 2026-09-25

**Authors:** Xiaoling Chen, Ke Wu, Bo Zhu

**Affiliations:** 1 Obstetrical Department, Quzhou Maternal and Child Healthcare Hospital, Quzhou, Zhejiang, China; 2 Prenatal Diagnosis Center, Quzhou Maternal and Child Healthcare Hospital, Quzhou, Zhejiang, China; 3 Reproductive Medicine Center, Quzhou Maternal and Child Healthcare Hospital, Quzhou, Zhejiang, China

**Keywords:** diamond-blackfan anemia 12, hydrops fetalis, intrauterine growth restriction, perinatal death, RPL15 gene

## Abstract

**Background:**

The heterozygous *RPL15* gene (OMIM*604174) variants are associated with autosomal dominant Diamond-Blackfan anemia 12 (DBA12, OMIM#615550). Fewer than ten *RPL15* variants have been reported to date, and they are commonly associated with hydrops fetalis, intrauterine growth restriction (IUGR) and neonatal anemia. Fetal demise attributable to the *RPL15* variant identified in our patient was the first documented case of perinatal death associated with *RPL15*-related DBA.

**Methods:**

Trio-based whole-exome sequencing (trio-WES) was performed on genomic DNA (gDNA) of fetal muscular tissues and parental peripheral blood samples. Mutant *RPL15* expression vectors pcDNA3.1 (+)-FLAG-*RPL15*-mut and control pcDNA3.1 (+)-FLAG-*RPL15*-wt mammalian expression vectors were constructed. Both vectors were subsequently transformed into HEK293 T cells. Assays measuring the relative expression of *RPL15* gene mRNA and RPL15 protein were performed. Actinomycin D was used to detect the degradation of *RPL15* mRNA. Meanwhile, we reviewed previously reported *RPL15*-related cases with available clinical data, including relevant genetic testing results and prenatal/postnatal manifestations.

**Results:**

Trio-WES identified a novel heterozygous frameshift *RPL15* gene variant (NM_002948.5) c.227del (p.Pro76Leufs*17) in this fetus. The relative expression of *RPL15* gene mRNA analysis showed that the mRNA expression of mutant RPL15 cell lines was significantly decreased, and the relative expression level of RPL15 protein in the *RPL15*-mut cell lines was significantly lower than that in the *RPL15*-wt cell lines (*P* < 0.0001). After actinomycin D treatment, mRNA transcription was inhibited in both the *RPL15*-wt and *RPL15*-mut cell lines; the *RPL15*-mut cell lines exhibited a significantly accelerated mRNA decay rate and lower mRNA expression relative to the *RPL15*-wt cell lines. All these results revealed that this variant resulted in a dramatic reduction in the formation of RPL15 protein due to the decay of mutant *RPL15* mRNA. After reviewing the current literature, we found that fetuses with *RPL15* gene variants commonly presented with hydrops fetalis, intrauterine growth restriction, and heart defect. The penetrance of loss-of-function variants in the *RPL15* gene was high, but appears to be incomplete.

**Conclusion:**

Fetuses with *RPL15* gene variants can present with hydrops fetalis, IUGR, heart defects, decreased fetal movements, and extremity abnormalities. Our functional studies have provided preliminary evidence confirming the haploinsufficiency of the *RPL15* gene as the underlying pathogenic mechanism of DBA12. This study also expands the mutational spectrum of the *RPL15* gene and broadens the associated clinical spectrum of DBA12.

## 1 Introduction

Hydrops fetalis (HF) is characterized by abnormal interstitial fluid accumulation within at least two fetal body compartments detected by ultrasound, including pericardial effusion, pleural effusion, ascites and skin oedema (Dalton et al., 2025). The pathophysiology of HF can be classified according to immune and nonimmune etiology. Nonimmune hydrops fetalis (NIHF) accounts for almost 90% of hydrops cases (Khairudin et al., 2023). NIHF is associated with a high perinatal mortality rate ranging from 50% to 98% (Turgal et al., 2015). A systematic review and meta-analysis revealed that the diagnostic yield of prenatal exome sequencing for NIHF was about 37% (Al-Kouatly et al., 2023). Herein, we present an extremely rare case of hydrops fetalis caused by a novel frameshift variant in the *RPL15* gene, resulting in perinatal lethality. The heterozygous *RPL15* gene (OMIM*604174) variants are associated with autosomal dominant Diamond-Blackfan anemia 12 (DBA12, OMIM#615550). The hallmark of DBA is haploinsufficiency of ribosomal protein gene, which disrupts ribosomal RNA (rRNA) maturation and stability of the ribosome. Impaired ribosome biogenesis triggers nucleolar stress, leading to TP53 stabilization and activation, which in turn induces apoptosis and disrupts differentiation of erythroid progenitor cells (Da Costa et al., 2024). Fetuses harboring *RPL15* gene variants commonly present with hydrops fetalis and IUGR. After reviewing the current literature, we found that fetal demise attributable to the *RPL15* variant identified in our patient was the first documented case of perinatal death associated with *RPL15*-related DBA. We also performed a series of functional studies on this variant, which preliminarily suggested that the pathogenic mechanism of DBA12 may be associated with *RPL15* haploinsufficiency.

## 2 Materials and methods

### 2.1 Whole-exome sequencing (WES)

Trio-based whole-exome sequencing (trio-WES) was performed on genomic DNA (gDNA) of fetal muscular tissues and parental peripheral blood samples using Genome Column Medium Extraction Kit (Kangweishiji, China) according to the manufacturer’s instructions. The extracted DNA samples were subjected to quality control using a Qubit 2.0 fluorimeter and electrophoresis with a 0.8% agarose gel for further protocols. The xGen™ Exome Research Panel v2 (designed by Integrated DNA Technologies) was used for WES. Quality control (QC) of the DNA library was performed using an Agilent 2,100 Bioanalyzer System (Agilent, United States of America). DNA nanoball (DNB) preps of clinical samples were sequenced on ultra high throughput DNBSEQ-T7 platform (MGI, Shenzhen, China) with paired-end 150 nt strategy following manufacturer’s protocol. For more details, please refer to the Supplementary material S1 (the process of whole-exome sequencing analysis) and 2 (a comprehensive list of candidate variants).

### 2.2 Bioinformatic analysis

Sequencing data were analyzed according to our in-house (Chigene Translational Medicine Research Center) procedures. Raw data were processed to remove adapters and filter out low-quality reads. The trimmed reads were then mapped to the University of California at Santa Cruz (UCSC) GRCh37/hg19 reference genome using Burrows-Wheeler Aligner (BWA) software (version 0.7.17). The Genome Analysis ToolKit (GATK) software (version 4.2.1.0) was used for single nucleotide polymorphisms/variants (SNPs/SNVs) and short insertions/deletions (INDELs) (<50 bp) calling. Samtools (version 1.22.1) and Picard software (version 2.22.1) packages were used to generate high-quality binary alignment map (BAM) data by removing duplicate reads. CNV detection from WES data was performed using the Multi-Scale.

Reference (MSR) algorithm built into NxClinical version 6.1 software (Bionano, San Diego, CA). Variants were annotated for analysis using the single nucleotide polymorphism database (dbSNP, version 5.3), gnomAD exomes database (version 4.1.0) and Chigene in-house minor allele frequency (MAF) database. Tools of pathogenicity prediction like REVELAL and AlphaMissense were used for predicting possible impact of variants. As a prioritized pathogenicity annotation to the American College of Medical Genetics and Genomics (ACMG) guidelines, Online Mendelian Inheritance in Man (OMIM), Human Gene Mutation Database (HGMD) and ClinVar databases were used as conferences of pathogenicity of every variant.

### 2.3 Variants classification

The result of filter process of WES: thirteen variants in 12 genes were identified through thorough analysis of all genes for putative dominant disease-causing variants. Only one variants in one genes were associated with this patient’s phenotypes of hydrops fetalis (HP:0001789). The excluded 11 genes were as follows: *NFKB2*, *APOA4*, *ARHGEF10*, *CHD6*, *DVL3*, *GALK1*, *LILRB2*, *MBD4*, *ORAI1*, OSMR, *PDX1*.

All of these rare phenotype-related variants were classified by following the guidelines of ACMG for interpreting sequence variants. Classification considered the position of the variant in the human genome, MAF, the pathogenicity prediction of variants, disease mechanism, clinical phenotypes, literature evidence, evolutionary conservation.

### 2.4 Variants verification

Fetal and parental gDNA samples was used for the verification of this variant. All reactions were carried out in a total volume of 20 μL using the Taq DNA polymerase. After confirmation of the size of the amplicons, the PCR products were purified by standard protocol. The reaction system was: 2 μL DNA (10 ng/μL), 1 μL forward primer, 1 μL reverse primer, 10 μL PCR Master Mix, 6 μL free RNA H_2_O. The following protocol of PCR: initial denaturation at 95 °C for 3 min, followed by 30 cycles at 95 °C for 30 s, 60 °C for 30 s, and 72 °C for 30 s, with a final elongation at 72 °C for 10 min. The PCR products were electrophoresed on a 2.0% agarose gel and were sequenced. The primers were as follows: 5′-AGT​GGC​AAG​TGT​GTC​AAA​GGT​T -3′ and 5′-GCT​CAC​CTA​AGT​ACA​ATC​AGA​AAA​GT-3′. Sanger sequenced using the BigDye Terminator v3.1 Cycle Sequencing Kit (ABI 3730 Applied Biosystem, United States of America) for the verification of candidate variants. The identification of this variant was performed using Chromas software (version 2.2.6, Technelysium, Australia).

### 2.5 Construction of the *RPL15* variant *in vitro* and transfection

The coding DNA sequence *RPL15* gene is 615 base pairs. Mutant pcDNA3.1 (+)-FLAG-*RPL15*-mut, wild-type pcDNA3.1 (+)-FLAG-*RPL15-*wt and negative control empty mammalian expression vectors were constructed and purchased from Wuhan Biorun Biosciences Co., Ltd. Human embryonic kidney (HEK)-293 T cells were transfected with the above three expression vectors in separate tubes (one for the mutant expression vector, one for the wild-type expression vector, and one for the empty control vector). The constructed expression vectors were verified using Sanger sequencing. Then, 6 hours after the transfection, we gently removed the lipofectamine-containing medium, replaced the medium with Dulbecco’s modified Eagle medium (DMEM) containing 5% fetal bovine serum (FBS), and incubated the slide at 37 °C in a 5% CO_2_ incubator until day 2. On day 3, the samples were collected and used in a reverse transcription-polymerase chain reaction (RT-PCR) to quantify the *RPL15* mRNA expression and the Western blot (WB) assay to quantify the RPL15 protein expression. Each cell line underwent three independent replicates of the RT-PCR and WB assays. The RT-PCR and Western blotting experiments were conducted in accordance with the manufacturer’s instructions.

### 2.6 Reverse transcription-quantitative polymerase chain reaction (RT-qPCR)

Wild-type (*RPL15*-wt), mutant (*RPL15*-mut) and empty vector negative control expression vectors were respectively transfected into HEK293 T cells. All samples were collected at 24 h, 48 h, and 72 h post-transfection for subsequent analysis. The transfected cells were collected by centrifugation, followed by total RNA extraction using the Trizol method. Total RNA concentration and quality were checked using a NanoDrop 2000 spectrophotometer (Thermo Fisher Scientific, United States of America). Complementary DNA (cDNA) was synthesized using NovoScript® Plus All-in-one 1st Strand cDNA Synthesis SuperMix (Novoprotein, China) following the manufacturer’s instructions. The cDNA concentration was about 1,200 ng/μL. The total reaction system was 20 μL, with 500 ng of total RNA used as a template. Quantitative PCR was performed using the Maxima SYBR Green/ROX qPCR Master Mix (2×) (Thermo Fisher Scientific, United States of America). Relative *RPL15* mRNA expression levels were determined using 2^−ΔΔCt^ method, and the *ACTB* gene was used as the reference gene. The experimental values were normalized to the empty-24 h condition, designated as the internal control. The qRT-PCR reaction was performed in triplicate, and mean values and standard deviations were calculated from the obtained data. The qPCR primer sequences for *RPL15* were 5′-TTT​CTT​CTG​AGG​GTC​CGC​TG-3′ and 5′-ACG​TAA​CCT​TGC​TTG​GCC​TT-3’. The qPCR primer sequences for *ACTB* were 5′-CAT​GTA​CGT​TGC​TAT​CCA​GGC3′ and 5′-CTC​CTT​AAT​GTC​ACG​CAC​GAT-3’.

### 2.7 Western blot (WB)

Wild-type (*RPL15*-wt), mutant (*RPL15*-mut) and negative control empty expression vectors were separately transfected into HEK293 T cells. All samples were collected at 24 h, 48 h, and 72 h post-transfection for subsequent analysis. Western blot analysis was subsequently performed using standard protocol. Equal amounts of protein were resolved by sodium dodecyl sulfate-polyacrylamide gel electrophoresis (SDS-PAGE) and subsequently transferred onto polyvinylidene fluoride (PVDF) membranes. Membranes were blocked with 5% non-fat milk in a tris buffered saline with tween-20 (TBST) solution for 2 h at room temperature. Anti-Flag (MBL, Japan, dilution ratio is 1:1,000) and anti-β-Actin dilutions were added respectively and incubated at 4 °C overnight. Horseradish peroxidase (HRP)-labeled secondary antibodies were added and incubated at room temperature for 1 h β-Actin served as the loading control and internal reference protein. The loading amount of WB protein was 10 μg/well. Blot bands were quantified by densitometry using ImageJ software (ImageJ2). Protein levels were normalized to β-Actin. The relative RPL15 protein expression levels are quantified by the fold change (FC) in the ratio of the grayscale between the RPL15 protein band and the internal reference protein band.

### 2.8 Transcription inhibition

Actinomycin D is an inhibitor of mRNA and protein synthesis. Actinomycin D can directly bind to the guanine groups of single-stranded or double-stranded DNA in the transcriptional initiation complex, inhibit the activity of DNA-dependent RNA polymerase, and interfere with the transcription process of DNA, thereby inhibiting mRNA synthesis. Actinomycin D (10 μg/mL) was used to analysis the degradation of *RPL15* mRNA. Wild-type (*RPL15*-wt) and mutant (*RPL15*-mut) expression vectors were respectively transfected into HEK293 T cells for 48 h, then treated with actinomycin D for 0, 4, 8, and 12 h. RT-qPCR was used to detect the expression of wild-type and mutant mRNA. Experimental data were normalized to the respective 0-h baseline measurements.

### 2.9 Statistical analysis

Multiple comparisons across these experimental groups were performed using the Holm-Šídák multiple comparisons test in Prism software (GraphPad Prism 10.6.0). *P* < 0.05 was considered statistically significant.

### 2.10 Literature review

We systematically searched the PubMed database for relevant published studies on the research topic using “*RPL15* gene” as keywords. The search time was from the establishment of the databases to 28 February 2026. We reviewed *RPL15*-related cases with availability of clinical data including relevant genetic testing results, prenatal/postnatal manifestations.

## 3 Results

### 3.1 Clinical features

A 28-year-old woman (gravida 0, para 0) at 31^+2^ weeks gestation presented to our hospital reporting decreased fetal movements that had persisted for 1 day. On the morning prior to admission, the pregnant woman reported a noticeable decrease in fetal movements. Examination performed on the same day showed that the fetal heart rate was within the normal range. The following day, color Doppler ultrasound (Mindray® Resonar9t with SC6-1U ultrasound transducer, Shenzhen, China) revealed no detectable fetal heart rate or fetal movements, suggesting intrauterine fetal death. The fetal Doppler ultrasound scan revealed the following fetal findings: fetal position: left occipital anterior (LOA); biparietal diameter: 74 mm (−1.5 SD); head circumference: 265 mm (−2.6 SD); adominal circumference: 339 mm (4.9 SD); femur length: 50 mm (−2.9 SD). Ultrasound scan (Figure 1) revealed fetal scalp edema with a maximum thickness of approximately 5.7 mm, right thoracic pleural effusion measuring approximately 9.3 mm in width, and fetal abdominal ascites measuring approximately 15.2 mm in width. The woman delivered the stillborn infant on the second day after admission. The birth weight was 2020g. The infant presented with generalized marked edema. The external genitalia were ambiguous (Figure 1). This primigravida with an unremarkable medical history denied consanguinity and exposure to teratogenic or hazardous substances. Routine first-trimester serum screening and non-invasive prenatal testing (NIPT) performed at our institution yielded normal results. No pathogenic microorganism nucleic acids were detected in the peripheral blood and amniotic fluid samples of the pregnant woman through microbiological testing (including nucleic acid amplification tests for bacteria and viruses); biochemical indicators of liver and kidney function, as well as 12-lead electrocardiogram results, were all within age- and gestation-specific reference ranges. The woman declined the pathological examination but agreed to genetic testing only, thus preventing the acquisition of other characteristic clinical information.

**FIGURE 1:**
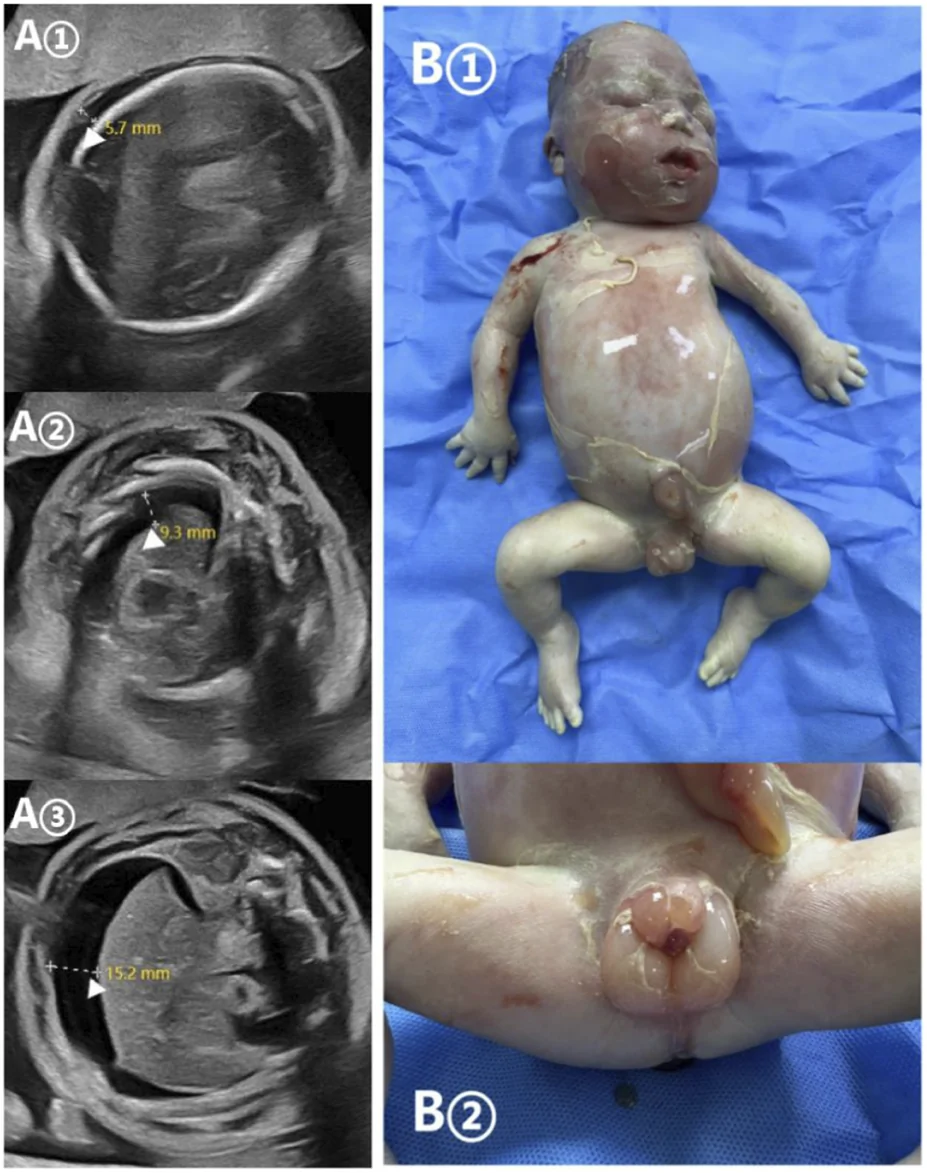
**(A)**. Doppler ultrasound imaging in fetus at 31^+2^ weeks’ gestation **(B)**. Postnatal images. B① edema throughout the body; B② ambiguous external genitalia.

A①: scalp edema with a maximum thickness of approximately 5.7 mm (indicated by the arrow).

A②: pleural effusion of approximately 9.3 mm wide in the right thoracic cavity (showed by the arrow).

A③: ascites of approximately 15.2 mm wide in the fetal abdominal cavity (showed by the arrow).

### 3.2 Genetic analysis and confirmation

Copy-number variants (CNVs) analysis of WES did not show abnormity. WES did not revealed pathogenic or likely pathogenic variants in genes associated with inherited metabolic disorders. WES identified a novel heterozygous frameshift *RPL15* gene variant (NM_002948.5) c.227del (p.Pro76Leufs*17), it was a *de novo* variant. This variant was further verified by Sanger sequencing (Figure 2). The allele frequency of this heterozygous variant has not been registered in population databases (1,000 Genomes Project, gnomAD, and dbSNP) or reported in disease databases (HGMD, OMIM) (PM2_Supporting). This fetus was the only *de novo* occurrence of this variant (PS2_Supporting). The probability of being loss-of-function (LoF) intolerant (pLI) value of *RPL15* gene in the gnomAD (v4.1.0) was 1.000, which indicated that *RPL15* gene was extremely intolerant to LoF variants (pLI>0.9). This variant in *RPL15* gene was not predicted to cause nonsense-mediated mRNA decay (NMD), the truncated region was critical to protein function (PVS1_Strong) (see Figure 2B, C). As per the interpretation guidelines by ACMG (Richard et al., 2015), this novel variant was classified as “likely pathogenic”. We have submitted this variant to a public database [Leiden Open Variation Database (LOVD)], and the accession number can be found at the following URL: https://databases.lovd.nl/shared/individuals/00468037.

**FIGURE 2:**
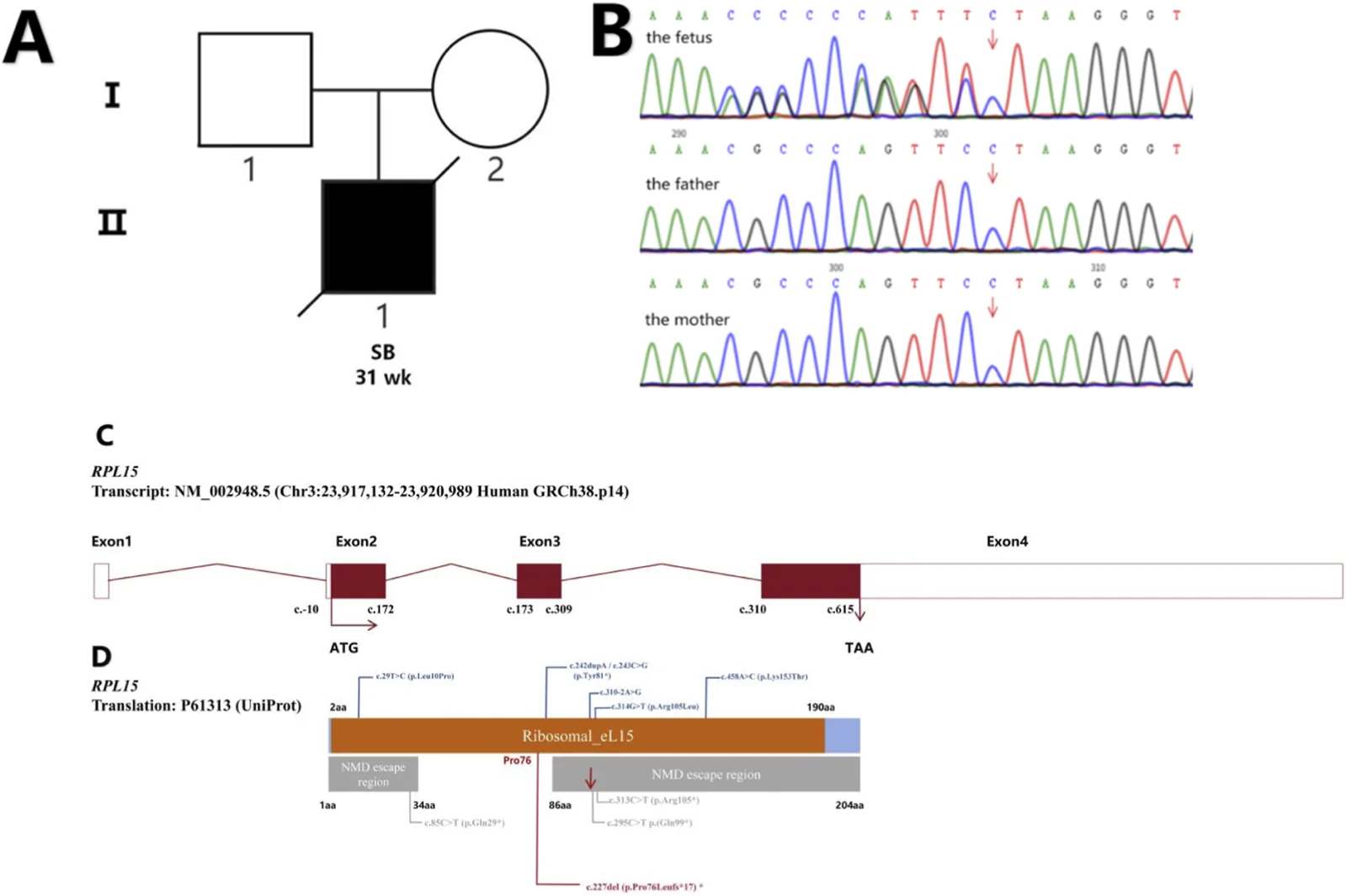
The pedigree, variant verification of Sanger sequencing and schematic diagram of the RPL15 gene transcript. **(A)** SB:stillbirth. wk: gestation week. **(B)** Sanger sequencing confirmed that the fetus carried the aforementioned novel variant heterozygous frameshift RPL15 gene variant (NM_002948.5) c.227del (marked by the red arrow), the parents were normal. **(C)** The transcript of RPL15 gene (NM_002948.5) has 4 exons (3 coding exons) with the transcript length of 2,155 base pairs, containing an open-reading frame (ORF) of 615 bp encoding 204 amino acids (aa). **(D)** Schematic diagram of ten RPL15 gene variants. According to the 50bp rule for predicted NMD escape, there are three NMD escape regions: ①first 100bp of coding exons (within 1∼34aa), ②last 50-54bp of penultimate exon (within 86∼103aa), ③last exon (within 104-205aa). The grey boxes mark NMD escape regions; the orange box marks critical protein domain “Ribosomal_eL15”; the grey lines mark the variants within NMD escape regions. The red line marks this novel variant reported in this study. The red arrow indicates the position of the premature stop codon.

### 3.3 Functional analysis of this frameshift variant

Sanger sequencing was performed to validate the full-length cDNA (NM_002948.5) of the *RPL15* gene in both wild-type and mutant cell lines. Sanger sequencing verified the successful construction of the mutant expression plasmid harboring the c.227del variant, prior to transfection of the overexpression plasmid (Supplementary Figure S1). To evaluate the effect of this frameshift *RPL15* variant, our functional studies revealed that this *RPL15* variant, c.227del, markedly decreased mRNA expression of *RPL15* gene (Figure 3), leading to a lack of normal RPL15 protein (Figure 3A, B) compared to the *RPL15*-wt cell lines.

**FIGURE 3:**
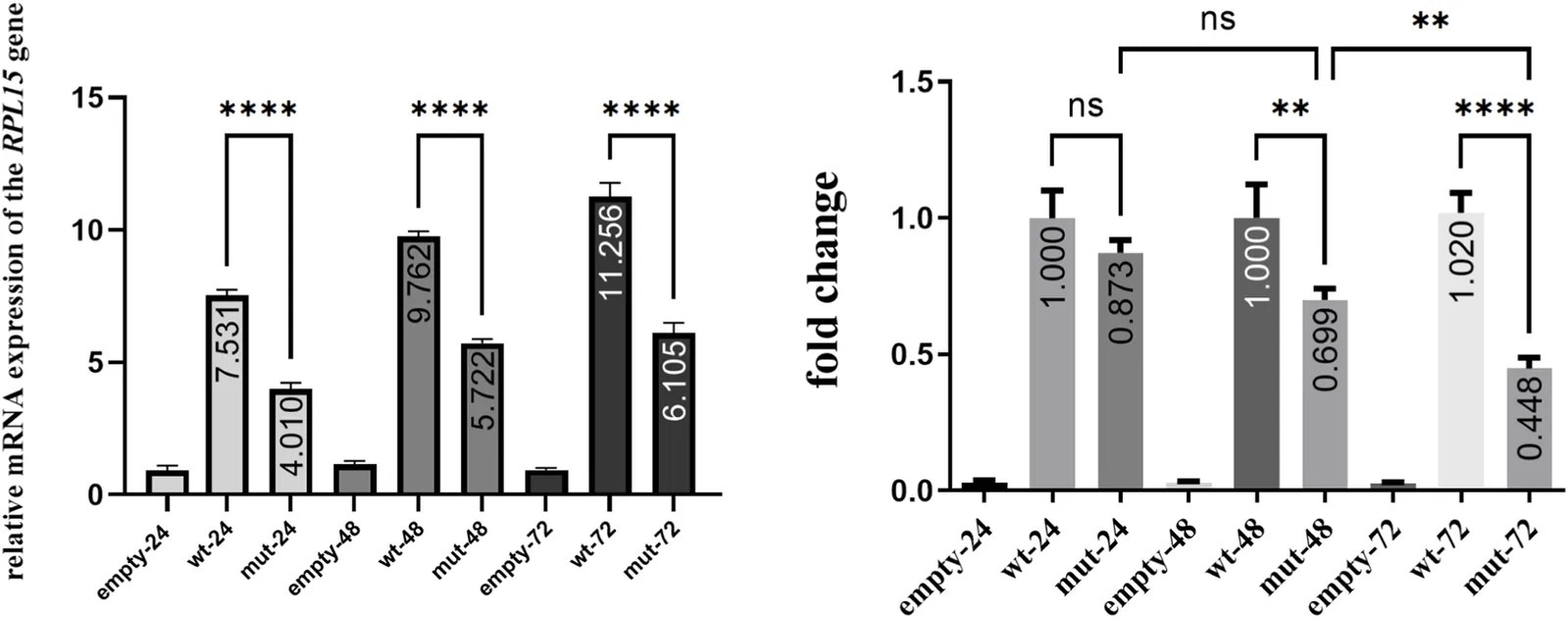
The relative mRNA and RPL15 protein expression.

As actinomycin D can bind to DNA to form a complex, hinder the function of RNA polymerase, and specifically inhibit mRNA synthesis, after actinomycin D treatment, RT-qPCR results indicated that mRNA transcription was inhibited in both the *RPL15*-wt and *RPL15*-mut groups; however, the *RPL15*-mut group exhibited a significantly accelerated mRNA decay rate and lower steady-state mRNA levels relative to the *RPL15*-wt group (Figure 4). This study hypothesized that this frameshift *RPL15* variant predicted to escape NMD may be subject to some certain mechanisms for degrading mutant mRNA.

**FIGURE 4:**
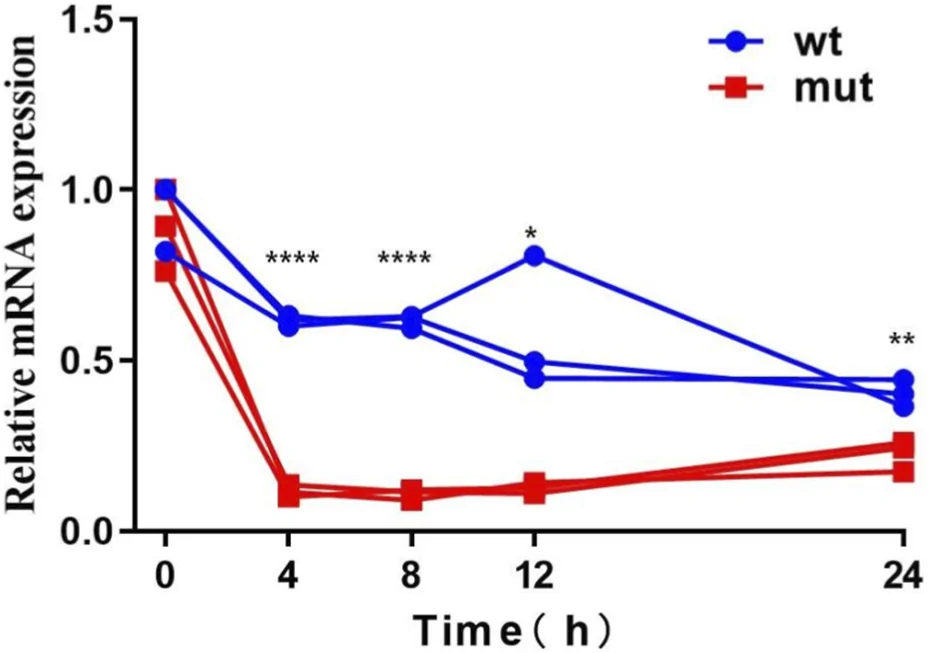
Following actinomycin D treatment, RT-qPCR results indicated that mRNA synthesis was inhibited in both the *RPL15*-wt and *RPL15*-mut groups, but the degradation rate was relatively faster in the *RPL15*-mut group. Experimental data were normalized to the respective 0-h baseline measurements. The relative mRNA expression levels of *RPL15*-wt and *RPL15*-mut groups at each time point were analyzed by independent sample Holm-Šídák test (****P < 0.0001, **P < 0.01, *P < 0.05). Each cell line underwent three independent biological replicates.

Based on these cases with frameshift/nonsense variants (eight cases) and deletions/intragenic deletions in the *RPL15* gene (three cases), we postulated that clinical phenotypes may be caused by LoF effects of *RPL15* variants and a molecular mechanism of DBA may be associated with haploinsufficiency of *RPL15* gene. The pLI value of *RPL15* gene in the gnomAD (v4.1.0) was 1.000, which indicated that *RPL15* gene was extremely intolerant to LoF variants (pLI>0.9). But the gene dosage sensitivity curation from ClinGen showed the little evidence for haploinsufficiency of *RPL15* gene (last evaluated time: 07/16/2014). This novel c.227del (p.Pro76Leufs*17) variant was a presumed LoF variant, which represented a putative mechanism underlying gene haploinsufficiency. Theoretically, this variant introduced a premature termination codon (PTC), resulting in premature translational termination and the production of a truncated protein comprising 91 amino acids (approximately 44.6% of the wild-type protein length). The 50bp rule for predicted NMD escape was first described by Nagy and Maquat (Nagy and Maquat, 1998). Validation of the exon junction complex (EJC)-dependent model and start-proximal NMD insensitivity were described by Lindeboom et al., 2016, (Lindeboom et al., 2016). If a PTC occurs close to the start or end of the transcript (NMD escape region defined as within the first 100bp of coding sequence, the last 50-55bp of the penultimate exon and the entire last exon coding sequence), then the corresponding transcript is predicted to escape NMD and thus avoids mRNA degradation. In accordance with this rule, this variant (c.227del) was located within the last 50-55bp of the penultimate exon coding sequence and was therefore predicted to escape NMD (Figure 2D).

3A: The experimental values were normalized to the empty-24 h condition, designated as the internal control. The results of RT-PCR analysis showed that the mRNA expression of mutant *RPL15* cell lines was significantly decreased. Compared with *RPL15*-wt cell lines harvested at 24, 48, and 72 h post-transfection, each cell line was subjected to three independent biological replicates of the RT-PCR assay. The difference between *RPL15*-wt and *RPL15*-mut was assessed using the Holm-Šídák test, with ****P < 0.0001.

3B: The relative RPL15 protein expression was quantified by the fold change (FC). WB analysis indicated that the relative expression level of RPL15 protein in the *RPL15*-mut cell lines was significantly lower than that in the *RPL15*-wt cell lines harvested at 48 and 72 h post-transfection. From 24 to 48 h, FC of *RPL15*-mut cell line showed a slow downward trend (increasing from 0.87 to 0.70), while from 48 to 72 h, it exhibited a more significant downward trend (decreasing from 0.70 to 0.45). Each cell line underwent three independent biological replicates of WB analysis. The difference between *RPL15*-wt and *RPL15*-mut was evaluated by Holm-Šídák test. **P < 0.01, ****P < 0.0001, ns: not significant. Note: this is FLAG (exogenous protein only) detection.

## 4 Discussion

The *RPL15* gene (ribosomal protein L15, OMIM*604174) is located on chromosome 3p24.2 (chromosome 3: 23,917,132–23,920,989 forward strand, GRCh38. p14). The transcript of *RPL15* gene (NM_002948.5) has four exons (3 coding exons) with the transcript length of 2,155 base pairs (Figure 5A). Its translation product consists of 204 amino acid residues (UniProt database accession P61313). The ribosomal protein L15 is a component of the large 60 S ribosomal subunit (Liang et al., 2020). The RPL15 protein is localized in the cytoplasm and plays structural and functional roles in ribosome assembly and protein synthesis. Functional role in the biogenesis of ribosomes and the process of protein synthesis. During the ribosome assembly stage, RPL15 protein works in concert with other ribosomal proteins and rRNA to promote the maturation of the 60 S subunit (Aspesi and Ellis, 2019). Overexpression of RPL15 protein has been observed in esophageal cancer (Wang et al., 2001), gastric cancer tissues (Wang et al., 2006), breast cancer (Ebright et al., 2020) and intrahepatic cholangiocarcinoma (Wang et al., 2025). Deletion of this gene has been observed in Diamond-Blackfan anemia syndrome patients. DBA syndrome is a bone marrow failure syndrome (BMFS) characterized primarily by erythroid hypoplasia, other non-hematologic manifestations, and an increased risk of cancer (Wlodarski et al., 2024).

We found that the gene-disease validity curation from Gene Curation Coalition (GenCC) showed the “strong” gene-disease validity of *RPL15* gene (last evaluated time: 04/01/2021). To date, the gene-disease validity of the *RPL15* gene has not been curated in the Clinical Genome Resource (ClinGen) database. The *RPL15*-related autosomal dominant Diamond-Blackfan anemia 12 (DBA12, OMIM#615550) has been reported in eight studies, encompassing a total of 14 patients aged 0–22 years. We retrospectively reviewed a total of 11 patients with heterozygous missense/frameshift/nonsense *RPL15* variants (SNVs/indels) and a total of three patients with deletions or intragenic deletions affecting the *RPL15* gene. Although all these patients were diagnosed with DBA, three of whom lacked detailed clinical documentation. The most prevalent shared clinical features observed among the 11 patients included congenital anemia presenting within the first 6 months of life (100%, 11/11), and three patients had undergone prenatal intrauterine transfusion. Other non-hematologic manifestations included developmental delay (DD) (27%, 3/11), intellectual disability (ID) (18%, 2/11), cerebral palsy (18%, 2/11), abnormalities of the cardiovascular system (36%, 4/11), dysmorphic facial features (36%, 4/11), renal malformations (18%, 2/11), abnormalities of the male genitalia (18%, 2/11). No significant differences in clinical phenotypes were observed between individuals harboring *RPL15* gene SNVs/indels and those with *RPL15* gene deletions or intragenic deletions. The penetrance of SNVs/indels in the *RPL15* gene appeared to be incomplete. Wlodarski et al., 2018, (Wlodarski et al., 2018) reported two variants (c.242dupA and c.29 T>C), each inherited from phenotypically unaffected parents; all affected offspring carrying the variants were clinically diagnosed with DBA. Dorn et al., 2021, (Dorn et al., 2021) reported one patient with the c.313C>T (p.Arg105*) variant inherited from the healthy 24-year-old mother with short stature (152 cm).

Among a total of 10 cases with reported prenatal examination records (including our case), we found the pregnancies of 9/10 cases were eventful. Among the nine eventful pregnancies, hydrops fetalis was observed in five cases (56%), fetal growth restriction in five cases (56%), fetal cardiovascular abnormalities in three cases (43%), and decreased fetal movements in three cases (43%). Other abnormal features were extremity abnormalities and placenta previa. All these patients reported in the literature survived, among which three patients had received intrauterine transfusions. In our case, the fetus developed hydrops fetalis and subsequently died in the perinatal period at 31 weeks’ gestation, presenting with ambiguous external genitalia and a normal chromosome karyotype of 46,XY. The lethality associated with the *RPL15* variants was recapitulated in the mutant zebrafish model. There is currently no published evidence linking the atypical phenotype of ambiguous genitalia to pathogenic variants in *RPL15* gene. Given that this finding has been observed in only two patients to date, a causal relationship cannot be established, and the association may reflect coincidental co-occurrence, differences in genetic background, or the influence of unidentified modifying factors.

We have conducted *in vitro* functional studies on this variant. In fact, we found that the regions predicted to escape NMD were not always consistent with the actual experimental results. RT-PCR showed that the mRNA expression of mutant *RPL15* cell lines was significantly decreased compared with that in the *RPL15*-wt cell lines at 24, 48, and 72 h post-transfection (Figure 3A). *RPL15*-mut cell lines produced truncated protein (Supplementary Figure S2). Western blot analysis revealed that the relative expression level of RPL15 protein in *RPL15*-mut cell lines was significantly reduced compared with that in *RPL15*-WT cell lines at 48 and 72 h post-transfection (Figure 3B). Following actinomycin D treatment, the *RPL15*-mut group exhibited a significantly accelerated mRNA decay rate and lower steady-state mRNA levels relative to the *RPL15*-wt group.

To sum up, we speculated that this mutant mRNA (predicted to escape NMD) remained subject to degradation, thereby reducing mutant protein synthesis and inducing haploinsufficiency of the *RPL15* gene, which underlies the pathogenesis of Diamond-Blackfan anemia. Furthermore, two nonsense variants (c.313C>T, c.295C>T) located downstream of this novel stop codon and one nonsense variant (c.85C>T) within the first 100bp of coding sequence (also predicted to escape NMD) have been reported in three patients with DBA (Figure 2C). The truncated region resulting from these variants overlaps with the evolutionarily conserved, functionally critical protein domain “Ribosomal_eL15” (domain source: Pfam; accession: PF00827). During the earliest assembly stages of the large 60 S ribosomal subunit, eL15—alongside its neighboring ribosomal proteins eL8 and eL36—cooperatively shaped the 25S/5.8 S rRNA domain I. Deletion of *RPL15* affects the assembly and stability of the ribosome and efficient protein production. *In vivo* depletion of yeast eL15 resulted in the deficit of 60 S subunits and the appearance of half-mer polysomes (Fernández-Fernández et al., 2023). Wang et al., 2025, (Wang et al., 2025) identified a variant c.242dupA (p.Tyr81*) in three unrelated patients diagnosed with DBA. Subsequently, several *in vitro* functional assays were performed. They found that the variant c.242dupA (p.Tyr81*) impaired pre-rRNA processing, reduced 60 S ribosomal subunit formation, decreased *de novo* protein synthesis, decreased the number of erythroid colonies, and delayed in differentiation, in addition to inducing increased apoptosis, TP53 stabilization, and p21 overexpression in *RPL15*-mutant hematopoiesis. Collectively, they were the first to propose the hypothesis that *RPL15* variants could cause haploinsufficiency and lead to a reduction in ribosomal protein levels.

*In vivo* knockdown the *RPL15* in human A549 cells decreased free 60 S ribosomal subunit while concomitantly increasing the abundance of free 40 S subunit (Luan et al., 2022). Downregulation expression of *rpl15* in zebrafish by microinjection of *rpl15* morphilino antisense oligo (Zhou et al., 2021) and knocking out zebrafish *rpl15* gene by CRISPR/Cas9 editing technique (Peng et al., 2021) showed that downregulation of *rpl15* inhibited the early hematopoietic development of zebrafish and the mutant *rpl15* produced abnormal phenotypes (developmental delay, smaller head, shorter body length, curved tail, pericardial edema and reduced hemoglobin synthesis in erythrocytes) similar to Diamond-Blackfan anemia, approximately 95% of the mutant zebrafish died within 7–10 days after fertilization. Integrating evidence from literature review, clinical case/variants characterization, *in vitro* cellular functional assays, and data from *RPL15* gene knockout animal models, we supports a preliminary and prudent conclusion that *RPL15* gene variants may exhibit dosage-sensitive pathogenicity.

In summary, we report herein the fetal death may be associated with a novel *RPL15* gene variant. This study is based on a single-family cohort; therefore, additional cases sharing analogous clinical and genetic profiles are thus required to rigorously validate the causal relationship between *RPL15* variants and perinatal death. Clinicians should prioritize close follow-up and multidisciplinary management for patients or fetuses carrying *RPL15* variants. The penetrance of LoF variants in the *RPL15* gene is high, but appears to be incomplete. Therefore, during prenatal diagnosis, genetic counseling for fetuses carrying pathogenic *RPL15* variants but without clinical phenotypes should be conducted with caution. Current literature and our *in vivo* functional analysis collectively indicate that *RPL15* haploinsufficiency underlies the pathogenesis of *RPL15*-associated DBA (Table 1).

**TABLE 1:** Summary of the clinical phenotypes of patients with previously reported *RPL15* gene variants.

*RPL15* variants (NM_002948.5)	Prenatal presentations	Primary postnatal presentations	References
Deletion (includes intron 3 and exon 4) unknown inheritance	NA	female, anemia at birth, ventricular septal defect, triphalangeal thumbs, responded to corticosteroid	(Landowski et al., 2013)
Deletion (includes complete *RPL15* gene) unknown inheritance	NA	diagnosed with DBA	(Farrar et al., 2014)
c.310–2A>G unknown inheritance	NA	3 years old, male, anemia at birth, DD, cerebral palsy, radioulnar synostosis, horseshoe kidney	(Mirabello et al., 2017)
c.242dupA (p.Tyr81*) inherited from the father with elevated eADA (1396U/lec)	IUGR	6.5 years old, female, anemia at 3 months old, spontaneous recovery after 1 transfusion at 6 months old, relapse at 5.7 years old, achieved remission after short course of steroids	(Wang et al., 2025)
c.242dupA (p.Tyr81*) *de novo*	hydrops fetalis	5 years old, male, ID, cerebral palsy, left cerebellar hypoplasia, ptosis, flat nose, deep set ears, intermittent AV-block, duplex left kidney, hypogonadism, intersexual genitalia (genetic test showed 46,XY), microcephaly, achieved remission after short course of steroids	
c.242dupA (p.Tyr81*) *de novo*	hydrops fetalis, IUGR, persistent foramen ovale	16 years old, female, 2 transfusions after birth, achieved spontaneous remission at 4 months old	
c.85C>T (p.Gln29*) unknown inheritance	hydrops fetalis	18 years old, male, hypogammaglobulinemia, plantar warts during steroid therapy, regular transfusions, transfusion-dependent from age of 9 years	
c.29T>C (p.Leu10Pro) inherited from the healthy mother	placenta previa	2 years old, anemia at 4 months old	
c.458A>C (p.Lys153Thr) unknown inheritance	Unremarkable	22 years old, male, anemia at 6 months old, ID, DD, low-set hair line, transfusion dependent from age of 5 years	
c.243C>G (p.Tyr81*) unknown inheritance	NA	diagnosed with DBA	(Ulirsch et al., 2018)
c.295C>T (p.Gln99*) Unknown inheritance	NA	diagnosed with DBA	
c.314G>T p. (Arg105Leu) *de novo*	IUGR	54 days old, male, respiratory insufficiency, pulmonaryarterial hypertension, pulmonicstenosis. Depressed nasal bridge, anteverted nares, long philtrum, narrow mouth, micrognathia, single transverse palmar crease	(Australian Genomics Health Alliance Acute Care Flagship et al., 2020)
c.313C>T (p.Arg105*) inherited from healthy 24-year-old mother with short stature (152 cm)	fetal growth restriction, abnormalities of the right upper extremity, biventricular hypertrophy, endocardiofibroelastosis, decreased fetal movement	male newborn, anemia, respiratory failure at birth, low-set ears, hepatomegaly, micropenis, abnormal pronation and absence of the thumb, patent ductus arteriosus with right to left shunting, atrial septal defect, severe pulmonary hypertension, and right ventricular hypertrophy	(Wlodarski et al., 2024)
Deletion (includes complete *RPL15* gene) unknown inheritance	decreased fetal movements, fetal heart rate tracing showed reduced variability, pleural effusion, ascites, subcutaneous oedema	female newborn, anemia at birth, DD, Dandy Walker malformation	(Dashraath et al., 2023)
c.227del (p.Pro76Leufs*17) *de novo*	decreased fetal movements, fetal growth restriction, pleural effusion, ascites, subcutaneous oedema	edema throughout the body, ambiguous external genitalia (chromosome karyotype showed 46,XY)	this case

IUGR: intrauterine growth retardation; ID: intellectual disability; DD: developmental delay; NA: not available.

## Data Availability

The original contributions presented in the study are included in the article/Supplementary Material, further inquiries can be directed to the corresponding author.
