# Supplementary material for: A novel frameshift RPL15 gene variant associated with hydrops fetalis and perinatal death
Source: Front Genet. 2026 Sep 25;17:1924019. doi: 10.3389/fgene.2026.1924019 (PMC13649215; doi:10.3389/fgene.2026.1924019)
Supplement: Supplementary file 1 [file Supplementaryfile1.docx]

**Figure 1:** The Sanger sequencing verification results of wild-type and mutant plasmids (showed by the blue box).

**Figure 2: Western blot analysis of RPL15 protein.**

The results of WB analysis showed that mutant *RPL15* cell lines could not produce the normal RPL15 protein compared to the *RPL15*-WT cell lines harvested at 72 hours post-transfection. Each cell line underwent three independent replicates of WB analysis.
