# Supplementary material for: A novel frameshift RPL15 gene variant associated with hydrops fetalis and perinatal death
Source: Front Genet. 2026 Sep 25;17:1924019. doi: 10.3389/fgene.2026.1924019 (PMC13649215; doi:10.3389/fgene.2026.1924019)
Supplement: Supplementary file 2 [file Supplementaryfile2.docx]

Method of whole-exome sequencing (WES)

1. Specimen collection: The EDTA-treated specimens were collected with informed consent of the patients tested.

2. DNA extraction: The umbilical cord blood or fetal tissue genomic DNA was extracted using the Blood Genome Column Medium Extraction Kit (Kangweishiji, China) according to the kit instructions. The extracted DNA samples were subjected to quality controlling using Qubit 2.0 fluorimeter and electrophoresis with 0.8% agarose gel for further protocol.

3. Whole exome library construction: Liquid hybridization of the genomic DNA was performed using xGen™ Exome Research Panel v2 (designed by Integrated DNA Technologies), and the target DNA fragments were enriched to construct exome library covering 19,119 genes with whole exons and partial introns. Each enriched region shared 40 Mb of targeted sequences.

4. Sequencing: High-throughput sequencing was performed by DNBSEQ-T7 platform (MGI, Shenzhen, China), and not less than 99% of target sequence were sequenced. The sequencing process was performed by the Zhiyin Oriental Translational Medicine Research Center.

5. bioinformatics analysis:

a.Quality control: Raw data were cleaned after adapters removing, low-quality reads filtering and other quality control protocol.

b.Variants calling: The clean data were aligned to the NCBI human reference genome (GRCh37/hg19) using BWA and variants were called using GATK. Samtools and Pindel were used to call SNPs (Single Nucleotide Polymorphisms) and indels, respectively. The clean data were than filtered, according to the quality of the sequencing, for further protocol.

c.Variants annotation and prediction: Nonsynonymous substitutions and SNPs with MAF (Minor Allele Frequency) lower than 5% were filtered using SIFT. The function of mutated genes and their pathogenicity were then analyzed referencing to dbSNP, 1000 Genomes Project, ExAC, ESP, OMIM, Swiss-var, HGMD, ClinVar and other disease databases.

d.The variants with unknown pathogenicity of single bases were screened by Provean, SIFT, Polyphen2-HVAR, Polyphen2-HDIV, Mutationtster and other protein structure prediction software. MaxEntScan were used to screen potential splice sites.
